# Blockchain for SME Clusters: An Ideation using the Framework of Ostrom Commons Governance

**DOI:** 10.1007/s10796-022-10288-z

**Published:** 2022-05-20

**Authors:** Geetika Jain, Archana Shrivastava, Justin Paul, Ronak Batra

**Affiliations:** 1 Keele Business School, Keele University, Keele, UK; 2 Jaypee Business School, Noida, Uttar Pradesh India; 3 Graduate School of Business Administration, University of Puerto Rico, San Juan, Puerto Rico USA; 4 University of Reading, Reading, United Kingdom; 5 Research Scholar, MDI, Gurgaon, India

**Keywords:** Blockchain, Small- and medium-sized enterprises (SMEs), Self-governance of communities, Technology Assessment, Clusters, Blockchain Technology, Blockchain Governance

## Abstract

Small and medium-sized enterprises (SMEs) organize themselves into clusters by sharing a set of limited resources to achieve the holistic success of the cluster. However, these SMEs often face conflicts and deadlock situations that hinder the fundamental operational dynamics of the cluster due to varied reasons, including lack of trust and transparency in interactions, lack of common consensus, and lack of accountability and non-repudiation. Blockchain technology brings trust, transparency, and traceability to systems, as demonstrated by previous research and practice. In this paper, we explore the role of blockchain technology in building a trustworthy yet collaborative environment in SME clusters through the principles of community self-governance based on the work of Nobel Laureate Elinor Ostrom. We develop and present a blockchain commons governance framework for the three main dimensions i.e., interaction, autonomy, and control, based on the theoretical premise of equivalence mapping and qualitative analysis. This paper examines the role of blockchain technology to act as a guiding mechanism and support the smooth functioning of SMEs for their holistic good. The study focuses on sustainability and improving productivity of SMEs operating in clusters under public and private partnership. This is the first study to address the operational challenges faced by SEMs in clusters by highlighting the dimensions of blockchain commons governance dimensions.

## Introduction

There is a growing interest in the governance of society and the economy. In this context, this paper examines the role of small and medium-sized enterprises (SMEs) in maintaining the pace of economies and ensuring the transition from underdeveloped and developing economies to developed ones (Isaksen, 2018; Kozonogova et al., 2019). Businesses form the backbone of a healthy and promising economy Lehmann & Menter, 2018; Rudskaya & Rodionov, 2017; Schepinin et al., 2018). To create an enabling environment for operations and coordination among these SMEs, they are often organized into clusters based on underlying similarities in terms of opportunities or challenges they might face (Terstriep & Lüthje, 2018; Todeva, 2006). Cluster agglomerations (Todeva, 2006) are interconnected and complementary based on similarity (Bembenek & Kowalska, 2016).

Cluster ecosystems spur national economic activity, contribute to regional development, attract investment, and create jobs. It also localizes the economy to leverage local resources, infrastructure, and land (Berawi, 2018; Berawi et al., 2019). The ecosystem that supports small and medium enterprises (SMEs) and entrepreneurial ventures is hybrid. They involve actors from academia, government, and industry agents, which are widely known as triple helix actors (Etzkowitz & Leydesdorff, 2000). The business exchanges and relationships between them are multifarious and complex (Agostino et al., 2015; Huggins & Johnston, 2010; Jack et al., 2008). SMEs operating in the cluster ecosystem have lost competitive advantages due to unfavorable environmental conditions (Gilsing, 2000; Lan & Zhangliu, 2012). This is primarily due to inefficient governance mechanisms and structures (Gilsing, 2000; Lan & Zhangliu, 2012). Previous research suggests that a cluster ecosystem increases SME efficiency (Kudryavtseva et al., 2020). However, these clusters are often prone to governance problems, mismanagement, and a lack of trustworthy rules of conduct that acts as a stimulus for ineffective cluster implications and less than optimal benefits for stakeholders across multiple dimensions.

Critical challenges observed concerning cluster governance range from adherence to contractual definitions to identity management to dis-intermediation (Gilsing, 2009; Andersson et al., 2004). These gaps in the premise of effective cluster management in SMEs invite appropriate research to establish trustworthy, transparent, and traceable governance mechanisms for these clusters (Balestrin & Verschoore, 2016). There are several studies on the mechanisms, innovation process in cluster governance, structure and actors involved in cluster governance (Berthinier-Poncet’s 2014; Hashimy et al., 2021; Mikhaylov, 2013). However, each of them lacks the perspective of the autonomous and self-governance phenomenon of management and governance of these clusters.

In the self-governance-focused context, a seminal Nobel Prize-winning study by Ostrom (1990), ideation of fundamental premises for establishing community self-governance principles are articulated. Among other things, the postulates discuss the mechanism for ensuring optimal resource sharing and balanced individual and group interests (Cumming et al., 2020). This seminal research also explores the need for communication rules and protocols developed by the community (Rozas et al., 2021). However, the postulates established by Ostrom serve as a guiding framework to address the solution premise. Nevertheless, an appropriate medium/technological intervention must address the concerns/challenges of SME clusters.

The recent technological advancement of Blockchain technology (Nakamoto, 2008) is a solution that offers perspectives of trust, transparency, and traceability in host systems with automated contracts that enforce compliance with business logic. Blockchain technology is an immutable ledger of data that relies on decentralization, non-repudiation, and disintermediation (Parekh et al., 2021). However, how the protocols that govern the blockchain evolve depends on the interplay of the actors involved (Pólvora et al., 2020). The trifecta of individuals, technology, and business entities engage and co-create, with the subsequent outcome of their overlapping engagements being the norms and values, which in turn will steer the relevance of blockchain governance (Dey et al., 2019; Liu et al., 2021).

It is daunting for MSMEs, facing intense competition in an increasingly global world. Not using technology to optimize business processes contributes to this and reduces the ability to compete in the global world (Mukherjee, 2018). In light of these challenges, the United Nations also promotes trade clusters and established the United Nations Industrial Development Organization (UNIDO) to enable MSMEs to become competitive and build their network (Bierce, 2019). Liu and Jiang (2020) proposed a blockchain-based decentralized and self-organized mechanism for MSMEs in the manufacturing sector. Researchers (Abou-Nassar et al., 2020; Chen, 2018; Wong et al., 2020; Choi et al., 2020; Nayak & Dhaigude, 2019) have mainly focused on the application of blockchain in supply chain. Researchers have historically focused on upgrading the technology to optimize the manufacturing process and quality production.

Clusters have to deal with a number of complex issues, such as active participation of institutions, technological innovation and capabilities, research and development, and close competition between MSMEs (Knorringa & Nadvi, 2016). To make clusters competitive, a system that provides institutional support based on trust is required to make clusters competitive (Humphrey & Schmitz, 2002). Therefore, the authors propose blockchain interventions to govern clusters.

While Ostrom’s principles, blockchain, and MSME clusters have been studied independently, there has been no effort to study their intersection. This study addresses this research gap by examining how blockchain can help MSMEs govern clusters based on Ostrom’s principles. In this study, we aim to explore how these three different concepts can come together to effectively manage clusters.

The premise of establishing an equivalence between the challenges/requirements of clusters in SMEs, Ostrom’s self-governance principles, and the offerings and artifacts of blockchain technology is a unique research premise that can be a guiding mechanism for further research and practice at large. Therefore, developing a comprehensive theoretical framework for cluster governance that leverages blockchain technology and Ostrom’s principles for effective cluster governance is of great importance. As the Blockchain phenomenon continues to rise, its sustainable form can only be manifested through a coherent contribution from both the technological and social fronts. Neither technology nor society can exist in a silo, and their engagement ensures that technology evolves based on societal needs and usage. This duality provides for an ever-evolving leap in progress that addresses the feasibility of a technological product (Orlikowski, 1992). The ever-expanding repertoire of applications of blockchain or Ethereum’s transition from proof-of-work to proof-of-stake are other examples of this duality (Sriman et al., 2021). The study aims to propose a governance framework for clusters challenged in the digital economy (Cassanego et al., 2019). The study offers theoretical explanations built on blockchain-based decentralized governance of clusters with governance rules defined in the blockchain. The premises for decentralized governance of clusters are based on Ostrom’s self-governance of communities. This paper addresses the two major shortcomings by building the ideating the meta-dimensions through a literature review on cluster governance and presenting the trifecta to establish the dimensions with the help of a qualitative analysis among three important aspects. Thereby, using this research approach and agenda (Beck et al., 2016) in the blockchain-governance solution, the critical research questions that we address in this study are as follows:

- How can these requirements be implemented in a blockchain-based architecture?
- Identify the challenges for cluster governance in SMEs.
- Formulate the equivalence of the theory of self-governance in communities to mitigate the challenges of SMEs and further transfer them mapping it further to the artefacts of blockchain technology.
- Rationalise the key dimensions of blockchain technology that contribute to SME cluster self-governance based on the guiding framework of Ostrom principles to formulate the blockchain commons governance framework.

The remainder of the paper is organized as follows. Section 2 introduces the complex perspectives of SME cluster governance, Ostrom’s self-governance principles, and blockchain technology. Section 3 provides a holistic overview of our research methodology. Section 4 presents the detailed analysis, and Sect. 5 discusses the proposed blockchain idea framework for SMEs. Sections 6, 7, 8, and 9 address the implications of the research, the contribution of the study, future directions, and the conclusion as the last section.

## Literature Background

### Blockchain Technology

Blockchain technology is the underlying technology of the successful cryptocurrency Bitcoin. However, with the advent of smart contracts and the vision of blockchain technology, the application realm has reached far beyond cryptocurrencies (Galvin, 2017; Parekh et al., 2021; Pawar et al., 2020; Jain et al., 2020). Haber and Stornetta ideated the foundation for blockchain technology by envisioning a ledger with a block design in which data is time-stamped and immutable (Haber & Stornetta, 1990).

Blockchain technology, as mentioned earlier, has applications in various fields, including society, governance, and business (Wang et al., 2018). Tech giants such as IBM have partnered with retail giant Walmart and logistics giants such as Merck to develop blockchain-based solutions that bring trust, transparency, and traceability to their systems (Androulaki et al., 2018; Galvin 2017). With this detailed and diverse understanding of blockchain technology as a tool for establishing trust, transparency, and traceability, we explore the utility of this technology for collaboration among SMEs within and across clusters.

Some of the key features of blockchain technology that can serve as a means to support SMEs in the cluster setting are summarized in Table 1.

**Table 1:** Blockchain technology feature

S. No.	Blockchain Technology Feature	Definition	Research Premise	BTF CODE
1	Data Immutability	Data once captured cannot be altered	(Azaria et al., 2016; Esposito et al., 2018)	BTF01
2	Incentive Mechanism	Reward though inbuilt cryptocurrency system	(Guadamuz & Marsden, 2015; Mehrwald et al., 2019; Swan, 2018)	BTF02
3	Decentralized	Involvement of stakeholders	(Cai et al., 2018; Kuo & Ohno-Machado, 2018; Patel, 2019)	BTF03
4	Non-Repudiation	Non-Denial	(Datta, 2019; Saxena et al., 2018)	BTF04
5	Disintermediation	Minimizing role of intermediaries	(Abe et al., 2018; Arya et al., 2019; Parekh et al., 2021)	BTF05
6	Confidentiality	Maintaining person and data confidentiality and anonymity	(Cong & He, 2019; Filippi & Hassan, 2018)	BTF06
7	Identity Management	Valid Identities Activation	(Hossain et al., 2018; Lone & Mir 2019; Ting et al., 2020)	BTF07
8	Simple Audits	Efficient validation	(Benchoufi & Ravaud, 2017; Kshetri & Voas, 2018)	BTF08
9	Smart Contracts	Logic Implementation	(Cong & He, 2019; De Filippi & Hassan, 2018)	BTF09
10	Consensus Mechanism	Incorporating Stakeholder Viewpoint	(Bach et al., 2018; Baliga 2017)	BTF10
11	BIoT	Blockchain and IoTfor real-time data	(Brandenburger et al., 2018; Hossain et al., 2018)	BTF11
12	BAI	Blockchain and AI for intelligent and trusted data insights	(Chen, 2018; Mamoshina et al., 2018; Mashamba-Thompson & Crayton 2020)	BTF12
13	Trust and Transparency	Trust and Transparency of entity involved	(Beck et al., 2016; Hossain et al., 2018; Karamchandani et al., 2020)	BTF13
14	Traceability	Source and Chain Identification	(Feng et al., 2019; Parekh et al., 2021)	BTF14
15	Tokenization	Participatory investment in operations	(Alabdulwahhab, 2018)	BTF15

The Blockchain Technology features discussed above in the Table 1, although not exhaustive, are surely representative of the strengths of Blockchain Technology that can be leveraged to cater to the requirements of SMEs in a cluster setting.

### Blockchain Governance

However, blockchain governance is another critical dimension of technology assessment that needs serious consideration given the research context. The governance levels are the off-chain community, the off-chain development, and the on-chain protocol (Pawar et al., 2020; Singh et al., 2019). The off-chain community includes requirements elicitation and documentation to create a trusted ecosystem for SME clusters using Ostrom principles. Finally, the on-chain protocol involves the incorporation of standards and protocols in the format of blockchain technology artefacts, i.e., consensus mechanism, smart contracts, identity management, etc., as described in Table 1 of blockchain technology characteristics. Data management is also an essential dimension when measuring the volume and diversity for a corresponding on-chain and off-chain secured mechanism.

The premise of blockchain governance is based on how certain norms and values induced by the interplay of stakeholders (individuals, technology, and companies) are enforced on the pre- existing protocol. The fundamentals that drive the blockchain governance paradigm include (a) ownership (Di Ciccio et al., 2019; Xu et al., 2017); (b) control of access (Hardin & Kotz, 2019) and the transaction process (Rikken et al., 2019). Facets such as data storage are a critical cog for blockchain governance to function seamlessly (Reijers et al., 2018). In hindsight, such instruments facilitating blockchain governance act as a bridge between owners and agents who collaborate in regulating a system governed by an algorithm-based protocol. The immutable nature of blockchain governance ensures that the framework is designed to control transactions. Transactions are not susceptible to human error or potentially unethical behaviour to which traditional regulatory mechanisms are susceptible to an adverse situation. The pre-agreed protocol that facilitates the transaction can be viewed as an actor which exercises governance. It does so through capabilities such as the approval structure and voting system (Kavanagh & Ennis, 2019; Lesavre et al., 2020; Swanson, 2015; Xu et al., 2016). The pre-ordained protocol, that can validate transactions independently ensures that parties involved in the transaction cannot manipulate this mechanism (Alexopoulos et al., 2018).

### Ostrom’s principles - self-governance of communities

The actual work on commons (Gordon, 1954) and after that on collective action (Olson, 1965) described the behavioral dilemmas of collective action in social science research. The term “collective action” refers to the collective action of a group to achieve its common interest (Olson, 1965). Subsequently, Hardin’s (1968) dissertation ‘the tragedy of commons’ examined the nature of an individual interested in maximizing his or her utility, which leads to a reduction of the commons. Due to an individual’s homo- economicus nature, a significant conflict arises in the group, which leads to a collective action of depletion of the commons. As a result, the conflict of short-term interests leads to unsustainability and it becomes imperative to manage the entities of the commons through a structure of governance or regulation.

Given the failure to manage common-pool resources, Ostrom (1990) argues that the approach to solving the commons problem goes back to the work of Hardin (1968). The idea of rational behavior is not to cooperate in a particular way for mutual benefit; instead, it is a variety of self-organized practices that enable communities to fairly and sustainably manage common resources for mutual benefit (Ostrom, 1990). Nobel laureate Ostrom (1990) described each participant’s contribution to and from the commons as the part of the community that becomes increasingly complex as it grows. It is required to define the boundaries of successful management of the commons within the community (Ostrom, 1990, 2000) (see Table 2). In her work, she showed the possible conditions under which the community can manage the commons. In her approach, she illustrated requirements that an individual cannot act in isolation, nor can he or she work in the community solely out of self-interest. In doing so, she argued that it is important to develop common protocols and rules within the community to ensure sustainability. The originality of the community boundaries she defined can lead to the demise of the commons if one participant in the network achieves an individual benefit at the expense of collective resources. Ostrom (1990) examined the meta-analysis of various case studies and theorized a set of principles for commons’ governance (Ostrom, 1990, 2000, 2005).

**Table 2:** Ostrom Principles

Ostrom Principles	Definition
Clearly defined community boundaries	It defines the rights of access and privileges to the stakeholders within the network.
Congruence between rules, local needs, conditions of common goods	The locus of rules that governs the behaviour of commons may change based on local conditions
Ensure participation in modifying the rules	In order to have collective choice arrangement and modification, people should participate in the network who are affected by rules
Monitoring	Some individuals in the network are accountable for the rest of the individual due to their role of monitoring of behaviour
Graduated Sanctions for rule violators	If there is any conflict or change in the behaviour of an individual in the network, other members may find it against the rules
Dispute resolution mechanisms	Accessibility to the low-cost conflict resolution spaces
Local enforcement of local rules	Enforced rules in the network with the approval of higher authorities
Multiple layers of nested enterprises	The layers of an organization to address the issues that may affect the resource management in the network

These principles have clearly defined the nature of the commons and have also been adopted in various studies on governance for the commons network in the digital space (Hess & Ostrom, 2007; Fuster Morell, 2010). The process of reemploying the Ostrom’s principles in a different context and reanalyzing their potential in a new context refers to changing their relevance in a social-techno perspective (Forte et al., 2009). Rethinking the theoretical basis to reapply the principles in self-governed small- to- medium-sized irrigation systems can yield much higher outcomes than any conventional theory (Sengupta, 1991; Ostrom, 2002). This paper is about developing a governance mechanism for SMEs in a cluster through community self-governance of communities by exploring the possibilities of blockchain-based governance. This study has explored all the intricacies of blockchain concerning SMEs in the context of governance of commons.

### Cluster governance and their enforced actions

This research analyses the mechanisms that can facilitate efficient governance of clusters based on Ostrom’s 8 principles for how commons can be governed sustainably and equitably in a community. We attempt to focus on sustainability, improving the capability and productivity of SME operating in clusters by applying Ostrom’s principles using blockchain technology under a people public and private partnership. Clusters are business networks of enterprises that have spatial proximity, similar techniques for production, adopt similar marketing practices, similar knowledge, face similar challenges, and have similar opportunities (Terstriep & Lüthje, 2018; Todeva, 2006). As Gilsing (2000) proposed, the concept of cluster governance indicates that cluster governance is a collective action by individual members for a common goal that enhances adaptability in a changing environment (Lan & Zhangliu, 2012; Liñán et al., 2020). A cluster is an association of MSMEs and defined by territory and proximity that nurtures trust among them. (Bierce, 2019). A cluster is a collaboration of independent and interdependent MSMEs and supporting institutions (Lu, 2020). Clusters is an association of enterprises located in a geographical area, producing similar or complementary products or services using similar technology levels, adopting similar marketing practices and communication channels, and facing similar challenges and opportunities. These enterprises can be connected by common infrastructure such as laboratory, quality control testing, etc. To address their challenges. Government or MSMEs ministries identify such clusters and provide assistance for their development (MSMEs, 2016, September 30).

Typically, SMEs that operate in a cluster have no social network to collaborate and interact. This reduces the flow of knowledge and exchange of information (Storey, 2004). Cluster governance needs to play the role of a regulator, coordinator, and controller. Therefore, it needs to develop a strategic knowledge base for the cluster, therefore playing the role of social architect (Arikan, 2009; Maskell, 2017). Berthinier-Poncet (2014) emphasized the need for mutual trust and cooperation for the governance of a cluster. Kudryavtseva et al., (2020) suggested that when SMEs work in clusters, eco-systems are more effective. It also localizes economies in terms of utilizing local resources, infrastructure, and land (Berawi, 2018; Berawi et al., 2019). Regardless of the growing significance of the clusters, many issues obstruct them from performing at their optimum.

Efficient cluster governance requires commitment and collective actions of all the stakeholders. It must ensure and sustainable competitive advantage interest of the stakeholders (Andersen et al., 2006). Business exchange and relationships among them are multifarious and complex (Agostino et al., 2015; Huggins & Johnston, 2010; Jack et al., 2008). Efficient governance of such an eco-system is essential for smooth conduct of economic activities, cluster development, increasing productivity, and infusing innovativeness.

Each of the entities belonging to a cluster operates for their agenda to achieve their objective. It is crucial to unite the plans into a common objective that benefits an individual organization and an entire eco-system cluster (Meier zu Köcker & Rosted, 2010). The challenge is resolving the conflicts, reaching a consensus, and working collectively towards the common objective. It requires a certain degree of social trust, collaboration, support, and monitoring (Bembenek et al., 2016). The critical challenges of cluster governance are the participation of all the cluster members, their commitment towards the common cause, and transparency of the system for all the stakeholders, their accountability towards common activities, efficiently doing the work, responsiveness towards cluster objectives, equal rights to all the cluster participants, the system of reaching to an agreement by all the cluster members (Etzkowitz et al., 2008). Cluster governance’s importance cannot be ignored due to the complexities involved in the management and its significance in the economy’s growth (Balestrin & Verschoore, 2016).

## Research Methodology

Blockchain governance is at a nascent stage and is evolving continuously in an inter-organizational context. In this study, a systematic search was carried out for literature review and qualitative methods were used to get the responses from market practitioners’ interviews (Tricco et al., 2017). Firstly, this study has adopted the literature review analysis to gain deep insights into various dimensions of cluster governance. Secondly, equivalence mapping is theorized to present the trifecta to establish the relationship. The systematic literature review was conducted by adapting the theory review method (Campbell et al., 2014; Thomas & Tee, 2021; Tranfield et al., 2003). First, a comprehensive review was performed to extract the literature on two aspects: cluster governance and the other for blockchain governance. Blockchain governance is a relatively new field of study compared to cluster governance; therefore, the availability of published literature is somewhat limited compared to the well-theorized concept of commons’ governance. To ensure the complete extraction of published literature on the stated topic and to develop dimensions, the broadly used databases were accessed such as Scopus, Web of Science, ScienceDirect, and Google Scholar (Mongeon & Paul-Hus, 2016). The database extraction method was used in addition to the snowballing method (Wohlin, 2014) to find the related literature of the topic.

**Fig. 1:**
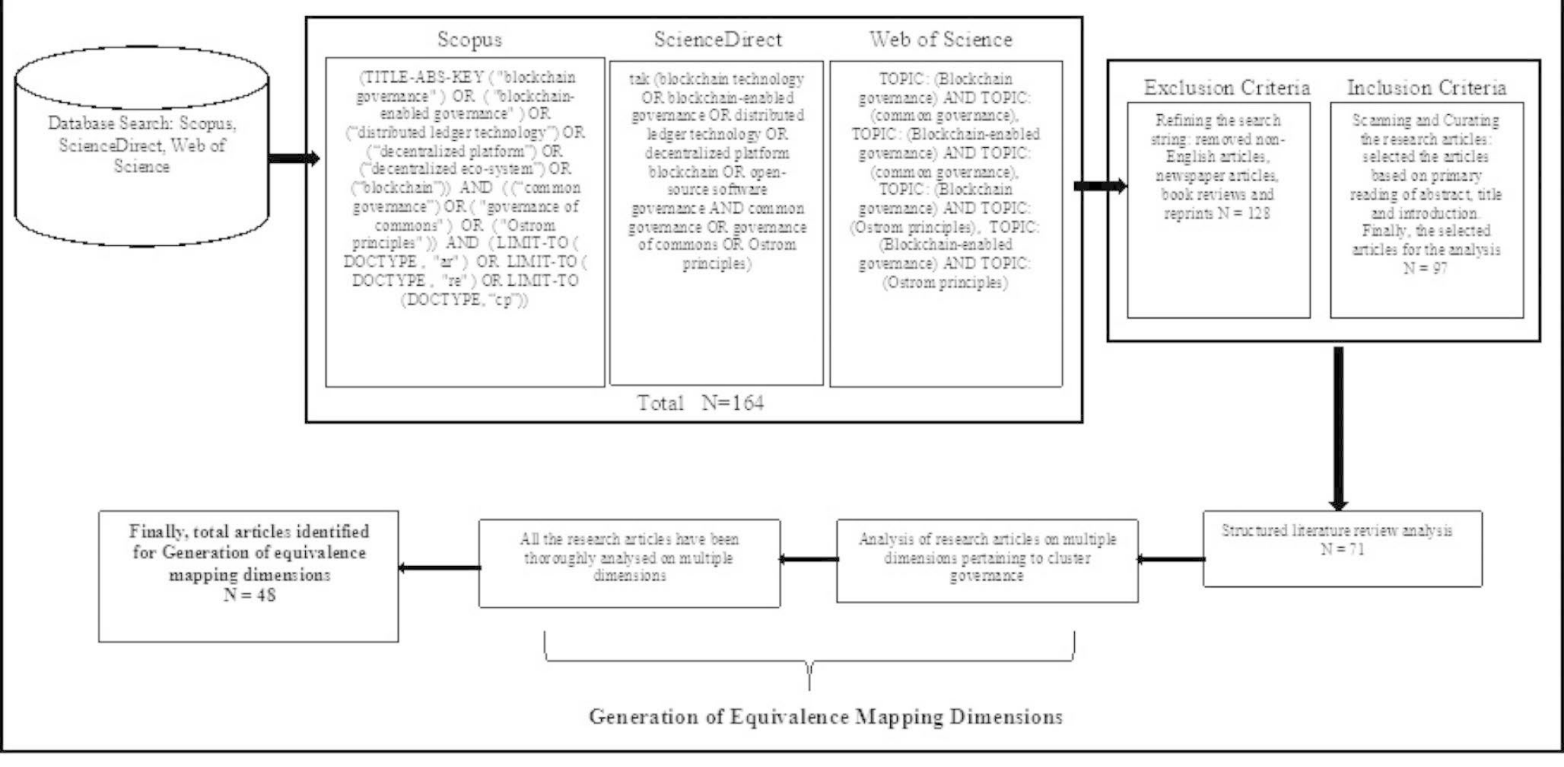
Systematic Literature Review

Further, both inductive and deductive approaches were adopted to trace the relevant theoretical perspectives and concepts (Fig. 1) (Clarke & Braun, 2014). The information base is used to develop the theoretical equivalence mapping among the trifecta. The equivalence mapping was used to create and explain the relationship between trifecta based on the identified concepts and their relationship (Bhattacherjee, 2012).

### Review of literature

To address the stated research questions, a systematic search for literature was carried out as the first step. To structure and synthesize the search output, the systematic search for literature is an appropriate technique to get the results on the published literature (Claire et al., 2020; Petticrew, 2001). In the systematic search for literature process, further steps are adopted to find the publication on the blockchain technology only peer-reviewed high-quality journals publications.

#### Search Outcome

After extracting relevant literature, the dimension matrix with detail has been developed through literature to help understand the theoretical aspect of building a new framework, supporting the analysis within the literature premise Radu-Lefebvre et al., 2021; Ramdhani et al., 2014;). Later, the synthesized matrix lists different governance dimensions; these dimensions are overarching the critical fundamentals of governance that are important in cluster governance through blockchain governance. While generating these matrices, the overlapping and related governance dimensions were grouped based on their definition. After this reiterative process, the most updated and structured fundamental dimensions were generated for the governance aspect. Further, these dimensions are used to develop the semi-structured interview questionnaire to develop a meta-dimensional view for the social – techno aspect rather than the techno-social view in framing the argument of governance of clusters by equivalence mapping among the trifecta.

### Qualitative research

Further, the second step of the qualitative analysis was performed to develop a meta-dimensional view for the social–techno aspect. Finally, eight responses are collected from senior or middle manager professionals from different industries and detail for the same is provided (Table 3). Due to the global pandemic scenario (COVID-19) (Aengenheyster et al., 2017), the online method was adopted to collect the responses from the respondents. Semi-structured interviews have been conducted with the stated target respondents (Table 3).

#### Data Abstraction

All the selected papers have been analyzed based on the content published to synthesize the information (Elo & Kyngäs, 2008). Further, the semi-structured questionnaire has been designed based on the synthesized information to collect the required insights (Elo & Kyngäs, 2008). Before conducting the semi-structured interviews, all the guidelines were taken into full consideration, i.e., shortlist the candidates based on their expertise and subject knowledge, and understand their empirical subject knowledge (Louise Barriball & While, 1994; Turner, 2010). The questionnaire was divided into two major sections: central theme and follow-up questions (Krauss et al., 2009). All the interviews were carried out on the online platform; each lasted for an average of 15–20 min and detail for the same are provided in Table 3. All the participants were selected from different backgrounds like consulting, governance, and public services in the blockchain-enabled solution domain, including geographical regions such as Germany, Spain, Denmark, and London. The interviews were conducted in the Europe and UK region by looking at the intensity of blockchain application solution in different SMEs under the EU blockchain strategy (European Commission, 2020, October 28). After that, the feedback was recorded to perform the thematic analysis to drive the dimensions of equivalence mapping (Clarke & Braun, 2014). We have adopted this methodology to formulate “a tested useful model” (Van de Ven, 2007), followed by developing a grounded framework by fitting the equivalence mapping analysis. Such techniques can provide critical information in developing theoretical arguments, conceptualizing the model, and building the framework about governance as the final output as analysis (Urquhart, 2010).

**Table 3:** Description of the Interview Respondents

Respondent No.	Current Designation	Industry Sector	Expereince
1	Blockchain Developer	Research and Development	6 years
2	Blockchain Consultant	Public services	8 years
3	Project Manager	Consulting	5 years
4	Executive Director	IT Services	7 years
5	Associate Consultant	IT Services	3 years
6	Project Consultant	Regulatory Institution	3 years
7	Senior Manager	IoT Consultant	6 years
8	Solution Architect	Business Agency Consultant	8 years

## Analysis

Based on all the responses’ content analysis findings, Ostrom principles and cluster governance elements led to mapping both the mentioned concepts to the blockchain technology features (BTF) (Table 1). Organizing the aspects of cluster governance with Ostrom principles resulted in the eight categories with the specific BTF, as shown in Table 2. Based on the analysis findings, we have described the governance of commons by analyzing the ability of blockchain technology at hand (Orlikowski & Iacono, 2001) and their ability to govern the dynamic system. In studying a blockchain governance system through the lens of Ostrom’s governance of commons, the primary vital takeaways with this alignment of cluster dimensions adhering to Ostrom Principles enabled to garner critical insights into viable mechanisms through which blockchain technology can facilitate cluster governance. Table 4 describes the trifecta to establish the equivalence mapping between three significant aspects where the multiple dimensions interact with the stated principles, following Rozas et al., (2021), Ostrom’s principles of communities to delve into the innovative potential of blockchain technology, while blockchain technology provides the support for coordination efforts to the clusters.

### Interaction

Unlike market exchange transactions – only two parties are involved in the exchange – the cluster has multilateral property. The extent of interaction between the participants is highly complex, where the exchange of information happens among the participants at one point in time. When the resources shared by multiple participants act as homo-economicus, the collective action depletes the commons. Thereby, it becomes necessary to manage the participants sustainably.

According to a blockchain consultant, “*But the basic problem is underlying…basic problem is the lack of communication exchange. And what we are doing is at the end of the day, people simultaneously exchange in the first time.”*

It becomes crucial to building a common set of rules and protocol in terms of communication among the participants to ensure sustainability within the network. To build a seamless exchange of information and sustainability, the community participants should follow a common space to resolve their conflicts. So, it’s based on the Ostrom’s 6th and 7th principles, where conflict resolution mechanism can be enforced with some defined local rules to achieve the cluster’s governance. Such defined rule will be embedded in smart contracts while the code will run itself (Reijers et al., 2018).

According to a Project Manager, “*This gives you trust on the engagement side…. It’s simply the content sharing principle with the management of the system. It’s a matchmaking algorithm that we’re currently working on, allows you to do matches between both sites and at messaging. So this all works on the end of the day to engage people starting interacting”.*

Overall, there is a high level of lateralness of exchange among all the participants due to the multilateral nature of the network where multiple participants interact.

According to Blockchain Solution Architect, “*You can do that for group as well on simply taking something and doing it. So, this is why you want to share with somebody. This is getting just simply getting access to the network for communication to get the power. this information, rather than simply the important information will simply be shown in the end of the day and it’s getting transformed for an activity in the form of asset*.*”*

Thus, the extent of lateralness of interaction among the participants may be disputed but resolving this is necessary. Therefore, in the case of cluster governance, the importance of lateral interaction should comply to the set protocol and rule of code of blockchain governance within the premise of Ostrom 6th and 7th principle. So, it’s based on the Ostrom’s principles of conflict resolution mechanism of community governance; in this case, maybe rules are embedded in the code to define the rules and the consensus mechanism.

### Autonomy

In the clusters, there is a common range of hierarchy followed with in the organization to interact based on the authority between the agents. Our findings indicate that participants in the cluster tend to be high in terms of having an exchange of information, socialization and collaboration.

According to Project Manager, “*you have a private group with something like a classified system, you have to knock on the door to get in and see the entries and then see the OK. That is a good indication of the difference in terms of autonomy to agents.”*

While exchanging information, a certain level of autonomy will be there as part of embedded rules in the network. The smart contract will be embedded in the network to interact among the participants as a decentralized autonomous organization (DAOs) – a self-governed organization runs by a set of rules.

According to Blockchain Consultant, “*all the organizations will engage in the transaction using smart contract. Whatever assets, you have digital efforts, you can tokenize out trading, that is something you can do with everything. Because you simply have to create your token, just put it on a cerium, put a value on it, and then this trading thing. And then the smart contract in the end of the day, organise how you want to handle, you can slice and dice a doll or whatever with the transaction in the network of clusters.”*

A token is an essential feature of blockchain, and it refers to the process of acting on an asset. Overall, blockchain technology can deploy tokenization to provide the complete authorization of information to all the participants in a distributed manner to gain incentives. In the network, DAOs will be fully autonomous and will hold tokens & assets. Thereby, DAOs will work based on the embedded code to fulfill Ostrom’s principles of 4th, 5th, and 8th (Monitoring, Graduated sanctions, and Multiple layers of nested enterprises) for cluster governance.

According to an Associate consultant,“*but as I said, the smart contract is not a contract. Simply holding, you’re simply holding in. You’re simply holding the the measures of what happens when, in the end of the day in the governance of organization specially SME. Yes, it’s it’s all about the smart contract And like token on the block and it’s going to provide the monetary value in the interest of the action.”*

Certainly, communities may have automatized processes using blockchain technology to accelerate the operation and reduce the burden of governance in the network. Autonomy plays a crucial role in blockchain for eliciting the behavior of network participants for maintaining the governance of clusters.

### Control

There is a series of autonomy goals of the participants within the network, which can be described as creating goals. By employing the rule in the network, Ostrom’s 1st principle was re-interpreted as the digital boundaries in the context of self-organized communities. Cluster governance is to control the participants through the rule of code in the shared economy.

According to Senior Manager (IoT Consultant),“*In my opinion, system are robust and transparent. Still, there’s not much clarity about the control of data, especially about regulating organisations in the network. And for this one, you need an community harmonisation. I think it has taken us a couple of years to come to this level of harmonisation become the half that is still not sufficient. And finish or Yes, it could do it, but it’s rather a power thing.”*

There is always a demarcation of power between local rules by the local authority and state institutions, commonly referred to as higher authorities. The rules are embedded in the code’s form to execute the control mechanism as an underlying technology. According to an Executive Director (IT service),*“Even before governing, because you’re always you always have a communicating, there’s blockchain technology background, you are, and you’re sure that everything you’re sharing is under certain terms and conditions, and it cannot be a new way of governance. So it has to be the end of the day.”*

To foster the rule of code as a type of agreement through blockchain technology, the organizations exercise the consensus mechanism embedded in a smart contract in the network instead of third-party rules. Furthermore, Ostrom’s 1st, 2nd and 3rd principles (clearly defined community boundaries, congruence between rules and local conditions, and collective choice arrangements, respectively) incorporate the self–management of resources through the blockchain governance in a cluster. Thereby, the rules are enforced by the code in the network to govern the cluster within community dynamics. So, the blockchain governed community will be controlled by the embedded set of rules to practice in the network.

## Discussion

Due to the unavailability of literature, it is necessary to develop a comprehensive theoretical framework for cluster governance that uses blockchain technology and Ostrom’s principles for efficient cluster governance. It is a far departure from totalitarian governance, which is evident in conventional governance systems in place. The rationale of interweaving blockchain technology and Ostrom’s principles in hindsight are an interaction of technology and societal norms thereby exemplifying the duality prevalent in technology (Orlikowski, 1992). Also, the governance is a system wherein the stakeholders co-create the mechanisms and their alignment is vital to ensure adherence to the protocols. This transpires on two fronts: (a) between technology and human/institutional aspects and (b) amongst social stakeholders (institutions, individuals such as banks and customers over ease of use of a payment gateway). Such interaction is vital to ensure that the relevance is not lost and co-creation is embedded in the DNA of consequential norms (Soni et al., 2021). This study aims to propose a theoretical framework for the governance of clusters. The study offers theoretical explanations building on the blockchain-based decentralized governance of clusters with governance rules specified in the blockchain. The premises for decentralized governance of clusters are set on Ostrom’s self-governance of communities. To establish the governance mechanism, we have devised the content analysis to insight theoretically using a pluralistic strategy (Mingers, 2001).

The summary has resulted in the development of the mapping of common governance framework that supports the network’s stakeholders from the perspective of blockchain governance. Table 4 has discussed the summary of the relationships based on Ostrom’s (1990) principles to frame our analysis and the mapping of principles of commons with blockchain governance dimensions.

**Table 4:** Equivalence mapping

Ostrom Principles	Cluster Governance Elements/Dimensions	Blockchain Technology Features
Clearly defined community boundaries	Transparency- Making accurate and relevant information available to all the stakeholders.	Smart Contracts (BTF09), Identity Management (BTF07)
Congruence between rules, local needs, conditions of common goods	Accountability- Co-ownership towards common activities and responsibilities	Smart Contracts (BTA09), Consensus Mechanism (BTF10)
Ensure participation in modifying the rules	Participation- involves various types of organizations such as SME, entrepreneurial ventures, govt. organizations, big firms etc.	Consensus Mechanism (BTF10), Identity Management (BTF07), Decentralization (BTF03)
Monitoring	Effectiveness- Correct orientation towards vision, mission, objectives and outcomes of the cluster	Smart Contracts (BTA09), Data Immutability (BTF01), Consensus mechanism (BTF10)
Graduated Sanctions for rule violators	Responsiveness- ensuring that the cluster objectives and activities take care of the current as well as the future needs of all the stakeholders.	Identity Management (BTF07), Incentive Mechanism (BTF02), Tokenization (BTF15), Simple Audit (BTF08)
Dispute resolution mechanisms	Consensus- reaching to an agreement for the interest of the cluster.	Smart Contracts (BTA09), Consensus Mechanism (BTF10)
Local enforcement of local rules	Commitment- obligation towards collaborative efforts	Smart Contracts (BTA09), Non-Repudiation (BTF04),
Multiple layers of nested enterprises	Inclusiveness- All stakeholders are empowered equally.	Decentralization (BTF03), Smart Contracts (BTA09), Consensus Mechanism (BTF10)

An overlap was found between Ostrom principles and cluster governance dimension endorsing participative decision-making (subject stakeholders such as SMEs and government institutions) concerning the formulation of relevant regulations.

### Extended Blockchain Commons Governance Framework

**Fig. 2:**
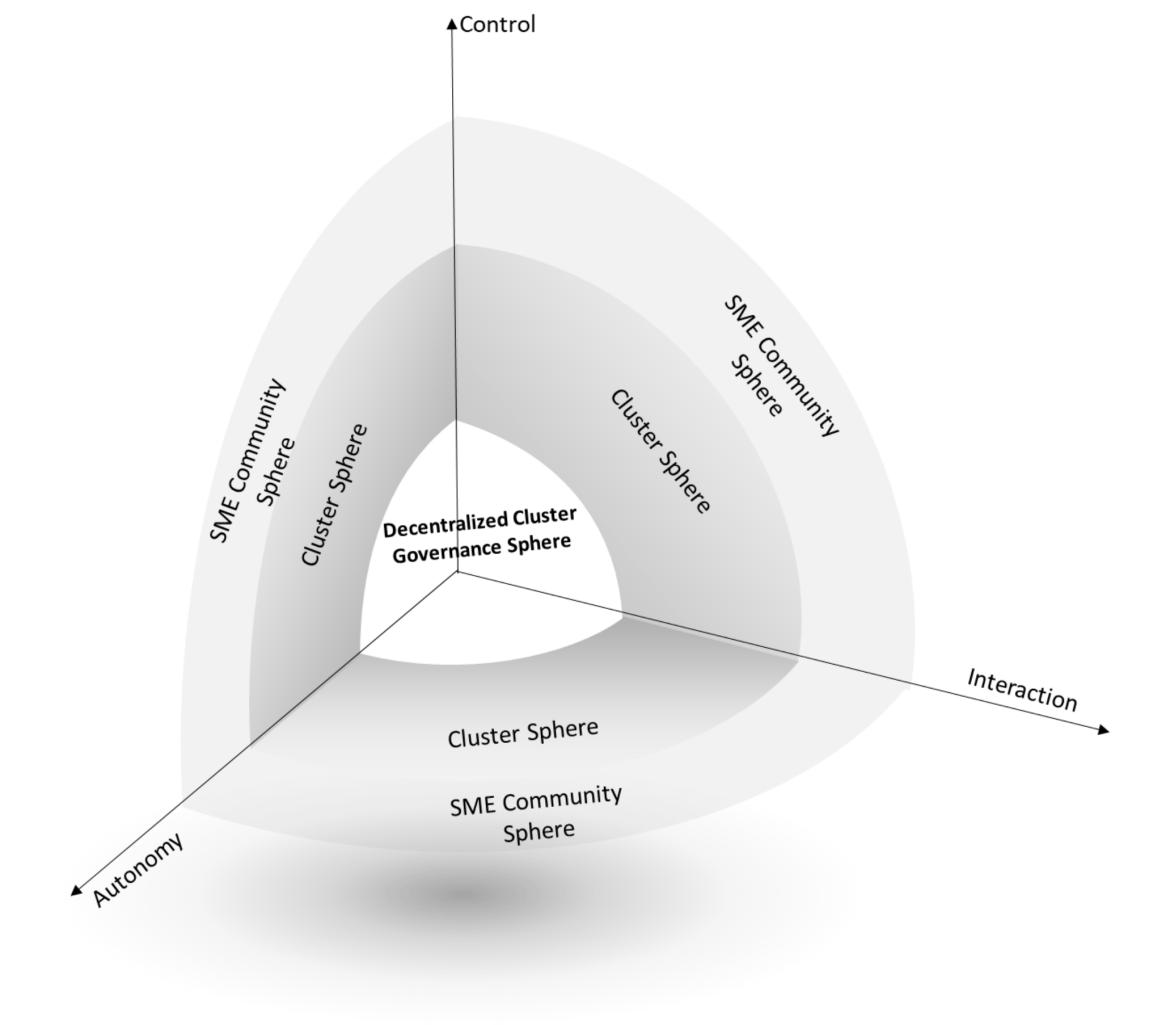
Extended Blockchain Commons Governance Framework

Further, equivalence mapping was employed to establish the trifecta and its dimensions of cluster governance using blockchain technology from the perspective of market practitioners. At this stage of development, investigating from limited literature and early-stage responses on the prospect of technology, it is difficult to draw the potential of blockchain and describe who it will evolve in the future. Certainly, there is a possibility of evaluation of blockchain that might affect the governance of cluster. By juxtaposing the blockchain governance and the cluster governance and the blockchain commons governance from interaction, autonomy, and control (see Table 5). We continue with further detail on the three significant dimensions of blockchain commons governance framework as illustrated in Fig. 2.

**Table 5:** Blockchain Commons Governance

Dimension	Dimension Property	Blockchain Network	Response Indicators
Interaction	Extent of lateralness	In a network, the stakeholders tend to interact by exchanging and sharing information with different agent at once	• *Basic problem is the lack of exchange of communication.* • *It’s simply the content sharing principle with management of the system. It’s a matchmaking algorithm that we’re currently working on, allows you to do matches between both sites and at messaging.* • *The access to the network for communication to get the power.*
Autonomy	Level of autonomous	To maintain the high degree of autonomy in the network, the central task is to examine each broad segment of blockchain commons governance	• *To have a classified system, it is a good indication of the difference in terms of autonomy.* • *The organizations will engage in the transaction using smart contract in terms of digital efforts*
Control	Control mechanism (local authority or state institution)	The degree of hierarchy within the network emerges based on the reputation and participant discretion	• *To have robust and transparent system, the clarity of the control is highly important.* • *If the technology is placed well, then the participants are sure about the sharing under those terms and conditions.*

The blockchain literature and the interview analysis suggest that the locus of interaction in the blockchain commons governance will be more digitalized and decentralized than the traditional approach of governance. Thus, the extent of lateralness underlines the genesis of this development. The essence of making interaction multilateral that proves the robustness and immutable nature of transactions ensures that the stakeholders have faith in blockchain transactions. Moreover, the governance itself is independent of the actions of stakeholders. Once a transaction is set in motion, the pre-agreed code dictating the smart contract will be executed, nonetheless. Our analysis illustrates that beyond the extent of lateralness, the control mechanism for an autonomous network is still at a nascent stage.

Blockchain commons governance might overlap between IT-enabled solutions for clusters and blockchain technology; there are considerable differences in governing the respective dynamics. Cluster governance facilitates improvement in the performance of SMEs clusters (Puppim & Jabbour, 2017). The literature review revealed various dimensions essential for cluster governance. These dimensions and elements are further categorized based on similarities identified in the content analysis. The study investigates the feasibility and usefulness of blockchain technology in the governance of clusters, and three dimensions are proposed to understand and analyze the governance of blockchain. Another unique aspect of the using blockchain technology for cluster governance is anonymity. The essence of blockchain governance is a far departure from the conventional governance mechanisms which are reliant upon institutions and their ability to enforce the regulations put in place (Li et al., 2010). The modus operandi of cluster governance using blockchain is relatively autonomous and relies upon protocols that have their genesis in formal coding languages. Unlike conventional transactions within the cluster network, where stakeholders are known to each other, parties collaborating in cluster governance enabled transaction are not aware of each other’s identity. The three major dimensions will make the whole governance system more robust in nature and ensure that the stakeholders have good faith in the whole governance system. In line with these dimensions, Fig. 2 shows the decentralized cluster governance sphere as the inner most part and describes the reliability of records stemming from twin tenets of immutability and ease of traceability ensures that blockchain as a governance mechanism can be trusted. Given this technology-enabled governance system, it can mitigate the various issues by ensuring that transactions deemed invalid will not be executed in the first place.

Given this technology-enabled autonomous discretion, it can mitigate the issues such as opportunism on the stakeholders involved. This illustrates that the blockchain provides a more robust system through the immutable nature of transactions, unlike conventional transaction systems, wherein the control mechanism will not be centrally placed. The reliability of records stemming from twin tenets of immutability and ease of traceability ensures that blockchain as a governance mechanism can be trusted.

## Research Implications

Our analysis of the governance of clusters through blockchain guided by Ostrom’s principles is theoretical. However, systematic review coupled with practitioners’ inputs provided a solid framework for further research. The study facilitates new perspectives on the application of blockchain in the sustainable governance of clusters. Hence, it enriches the existing literature on sustainable governance of SMEs clusters. The study will help to explore the potential of blockchain and Ostrom’s principles on the self-governance of clusters.

From the practitioner’s perspective, the study will be helpful for government and SMEs clusters to formulate strategies and prepare a roadmap for implementing the blockchain technology and Ostrom’s principles for self and the sustainable governance of SMEs clusters. A well-focused blockchain technology roadmap aids its successful adoption by SMEs clusters and will provide a sustainable competitive advantage. This trifecta presented is novel as it results from the thematic analysis carried out for equivalence mapping of blockchain, Ostrom’s principles, and cluster governance. The study is one of the first studies based on systematic literature review and semi-structured interviews of experts to generate the dimensions of blockchain commons governance. Blockchain commons governance framework presents a conceptual framework for using blockchain technology for SMEs clusters channelled by Ostrom’s principles. None of the previous research has carried out such in-depth research for the sustainable governance of SMEs clusters. Hence, the study also offers a valuable methodological insight into how these combinations of research methodologies can help develop insights on seemingly different concepts and theories and subsequently develop a solution to a research problem. .

## Contributions of the study

To facilitate a conducive environment of operations and coordination, the SMEs are often organized into clusters based on underlying similarities in terms of opportunities or challenges they might face. Every entity belonging to a cluster operates to achieve their own objective. The challenges faced by each entity while working together is resolving the conflicts, reaching a consensus, and working collectively towards the common objective. Social trust, collaboration, support, and monitoring is required within the cluster. The critical challenges of cluster governance are the participation of all the cluster members, their commitment towards the common cause, and transparency of the system for all the stakeholders, their accountability, efficiency, responsiveness towards cluster objectives, equal rights, the system of reaching to an agreement by all the cluster members. Although, there are a lot of complexities involved in the management, the cluster governance’s importance cannot be ignored. Blockchain technology is the underlying technology of cryptocurrency Bitcoin but now with the advent of the blockchain technology, this technology is not just limited to cryptocurrencies anymore. It now has applications in varied fields. The big tech companies like IBM have collaborated with retail companies like Walmart to bring blockchain technology in the retail sector with trust, transparency and traceability. This study aims to find the utility of this technology in the space of cooperation between SMEs in an intra and inter-cluster situation. This study has identified the challenges on cluster governance in SMEs and rationalized the key Blockchain Technology dimensions based on the guiding framework of Ostrom’s principles to aid self-governance of SMEs. The study focuses on sustainability, improving the productivity of SMEs operating in clusters under a people public and private partnership. This research investigates the governance of SMEs clusters through the adoption of blockchain technology. It shows that trifecta - interaction, autonomy, and control are the three pillars of decentralized cluster governance. The research presents a framework for SMEs governance and offeres directions for future research.

## Future research directions

SMEs serve as backbones of many economies, particularly emerging economies. A better understanding of the application and know-how of blockchain technologies for SMEs governance will need additional empirical research. The design and adoption of blockchain technology for cluster governance will require SMEs and other stakeholders such as the government and other organizations responsible for research and development, quality control, procurement of raw material, marketing etc. Table 6 summarized the research agenda that will help future researchers to investigate further in this area.

**Table 6:** Future Research agenda

Dimensions	Future Research Questions
Interaction	• How are interactions made in the blockchain commons governance? • How much the extent of lateralness impact the blockchain commons governance?
Autonomy	• How is autonomy determined in the blockchain commons governance? • How much the level of autonomous impact in the blockchain commons governance?
Control	• How is the control mechanism made in the blockchain commons governance? • How much the local authorities impact in the blockchain commons governance?

Further research can consider these stakeholders’ problems, views, and capabilities in adopting blockchain and Ostrom’s principles for governance. Also, further research can be conducted to have a deeper understanding of SMEs eco-system (following Chandra et al., 2020; Paul, 2020) to technological advancement and social practices that can be instrumental or can create potential hindrance in the adoption of blockchain technology and Ostom’s principles for self-governance. SMEs eco-system may also significantly vary in different cultures and countries and follow other practices. Hence, research on the applicability of Ostrom’s principles and blockchain technology in various cultural contexts is also an exciting area that can give some valuable insights.

Blockchain and Ostom’s principles may facilitate cooperation among SMEs in new ways. The amalgamation of Ostrom’s principles and blockchain technology will create a new pathway for the effective and sustainable governance of SMEs which is essential for the growth and economic development of a region. If implemented and adopted successfully, this study will open up new ways on how MSMEs function, collaborate and compete with each other.

## Conclusions

The congruence identified between Ostrom’s principles and cluster governance with that of blockchain technology gives directions to understand the scope of blockchain-based technologies in governing the clusters. In this study, the authors bring together the literature on the governance of SMEs clusters, blockchain-based governance, and Ostrom’s principles. The decentralized blockchain technology could enable coordination among SMEs. We presented potential blockchain features that may allow SMEs clusters to handle challenges associated with effective governance. Through this study, we see the opportunity in using the blockchain technology to increase the transparency and accountability.

Information system scholars may find the solution highly promising and they may use further and may bring deeply engrained phenomena of network technologies for SMEs. Indeed, blockchain technology has brought lot of attention in the academics to understand the dynamics of blockchain governance for SMEs. Therefore, this study tends to represent the use of blockchain technology as a new form of governance, thereby, the phenomena of blockchain for SMEs will surely effect the traditional form of governance. So, the scholarly discussions on blockchain for SMEs will unfurl the research gaps and may resolve the existing tension.

While, the academic research shed light on the new phenomena of using blockchain technology for SMEs, still, there is a lot need to discuss on the blockchain for SMEs from the market practitioner prespective. After pointing to several implication for academia, its important to unravel its importance for market practitioners and policy makers, this study conclude that blockchain for SMEs can change the way of doing business with in the cluster by incorporating more structured approach and could also enhance the understanding of organization dynamics within the working cluster. The study implies that the role of intermediaties might be complex in nature after the implementation of blockchain technology and the intermediaries can still play a complementroy role in order to perform various tasks including off-line assests verification and further digital form conversions.

It could also offer the better governance for the transacting partners within the cluster and may have strong relationships between different actors. Though, the lack of standardized regulations and institutional reforms may be the barrier in the implemation of blockchain for SMEs. Early discussions by the policy makers can suggest the further steps that can seek effective actions in response to this change. These influenced bodies can come up potential solutions that might trigger in future.

The current study has presented the SMEs clusters governance through blockchain technology through the three dimensions on the ostrom’s self governance of communities (Ostrom, 1990). Hence, the first and foremost limitation of this study is that the research question of this study is a reference point process and it may overlap with other studies conducted to develop the governance mechanism for a specific clusters. Second, it requires to formalize the rules of governance using blockchain technology because it is important that the machines need to understand the rules unambiguously. Thereby, its important to formalize the governance rules and encode them which presents a big limitation in the whole system.

The challenges faced by SMEs clusters are typically trust, transparency, and regulations; blockchain technology can tackle these challenges and, consequently, decentralize SMEs clusters’ governance. Past researchers have studied Ostrom’s classic principles and blockchain technology for the governance of Commons-Based Peer Production (Rozas et al., 2021). This study is focused on affordances in the context of Ostrom’s classic principles and blockchain governance. Calcaterra (2018) studied how blockchain technology can be applied in distributed autonomous organizations. Poux et al., (2020) analyzed blockchain application for the governance of common-pool resources. This research investigates the adoption of blockchain technology for the governance of SMEs clusters. It shows that trifecta - interaction, autonomy, and control are the pillars of decentralized cluster governance. We offer a research framework and agenda for SMEs governance in the network, and provide additional important possibilities for future research through critically examining the current theories present in the blockchain discourse.
